# Inhibition of PlexA1-mediated brain tumor growth and tumor-associated angiogenesis using a transmembrane domain targeting peptide

**DOI:** 10.18632/oncotarget.11072

**Published:** 2016-08-05

**Authors:** Laurent Jacob, Paul Sawma, Norbert Garnier, Lionel A.T. Meyer, Justine Fritz, Thomas Hussenet, Caroline Spenlé, Jacky Goetz, Julien Vermot, Aurore Fernandez, Nadège Baumlin, Samia Aci-Sèche, Gertraud Orend, Guy Roussel, Gérard Crémel, Monique Genest, Pierre Hubert, Dominique Bagnard

**Affiliations:** ^1^ Inserm U1109, MN3T Team, Strasbourg, France; ^2^ Université de Strasbourg, Strasbourg, France; ^3^ LabEx Medalis, Université de Strasbourg, Strasbourg, France; ^4^ Fédération de Médecine Translationnelle de Strasbourg (FMTS), Strasbourg, France; ^5^ Laboratoire d'Ingénierie des Systèmes Macromoléculaires (LISM), UMR 7255, CNRS-Aix Marseille Université, Marseille, France; ^6^ Centre de Biophysique Moléculaire, UPR 4301, CNRS, Affiliated to the University of Orléans, Orléans, France; ^7^ Institute of Genetics and Molecular and Cellular Biology (IGBMC), CNRS/INSERM/UDS, Illkirch, France; ^8^ Current address: Institut de Chimie Organique et Analytique UMR, Université d'Orléans, Orléans, France

**Keywords:** plexin, anti-cancer drug, angiogenesis, biomarker, glioblastoma

## Abstract

The neuropilin-plexin receptor complex regulates tumor cell migration and proliferation and thus is an interesting therapeutic target. High expression of neuropilin-1 is indeed associated with a bad prognosis in glioma patients. Q-RTPCR and tissue-array analyses showed here that Plexin-A1 is highly expressed in glioblastoma and that the highest level of expression correlates with the worse survival of patients. We next identified a developmental and tumor-associated pro-angiogenic role of Plexin-A1. Hence, by using molecular simulations and a two-hybrid like assay in parallel with biochemical and cellular assays we developed a specific Plexin-A1 peptidic antagonist disrupting transmembrane domain-mediated oligomerization of the receptor and subsequent signaling and functional activity. We found that this peptide exhibits anti-tumor activity *in vivo* on different human glioblastoma models including glioma cancer stem cells. Thus, screening Plexin-A1 expression and targeting Plexin-A1 in glioblastoma patients exhibit diagnostic and therapeutic value.

## INTRODUCTION

Glioblastoma (GBM) is a devastating disease with poor prognosis [1]. Highly infiltrative and vascularized, these tumors are composed of multi-clonal cell types with various migratory and proliferative properties, and are genetically very heterogeneous. This obvious cellular heterogeneity largely accounts for the observed resistance to all available therapeutic strategies including, surgery, radiotherapy and chemotherapy [2]. Significant recent progress in targeted therapy comprises blocking various receptor tyrosine kinases (RTK) [3] or inhibiting angiogenesis [4]. However, the clinical outcome remains poor and the overall survival of patients is only modestly improved over last decade [5]. Hence, there is a crucial need in identifying new therapeutic targets and to develop efficient inhibitors of these targets preferably avoiding side effects. We had taken advantage of one important requirement for signal transduction of RTK and other membrane receptors which is dimerization or oligomerization. We focused on the transmembrane domain of single-pass transmembrane proteins. The function of this domain contributes mainly to regulation of signal transduction rather than membrane anchoring [6]. Transmembrane domain interactions (TMD) have been described for many RTK including the ErbB family [7] and other cell surface receptors such as integrins [8], the amyloid precursor protein APP [9] or the T-cell receptor [10]. Thus, targeting TMD interactions represents a unique novel alternative strategy. Indeed, we had recently shown that the TMD of Neuropilin-1 (NRP1) is crucial for the dimerization and oligomerization of this receptor [11] that controls a wide range of biological functions. Strikingly, a synthetic peptide mimicking the TMD of NRP1 (MTP-NRP1) acts as a potent inhibitor by antagonizing dimerization. We had shown that MTP-NRP1 impaired tumor cell migration and angiogenesis both *in vitro* and *in vivo* assays thereby reducing brain tumor growth [12] thus suggesting that TMD-interfering peptides may represent a novel class of therapeutic agents [6]. Although most work had focused on homo-dimerization of TMD containing receptors, hetero-dimerization may be key to their wide signaling function. We decided to further explore the possibility of antagonizing signaling partners of NRP1 by interfering with hetero-association of NRP1 with other important cancer associated receptors.

Here, we report that Plexin-A1 (PlexA1), one of the signaling partners of NRP1 [13] is a potential novel prognostic marker for GBM patient survival. Using computer simulation and a two-hybrid system (BACTH) we further showed that NRP1/PlexA1 TMDs do interact with each other by forming trimers. We demonstrated that a synthetic transmembrane peptide mimicking the TMD of PlexA1 (MTP-PlexA1) reduced GBM cell proliferation and blocked VEGF-induced tumor cell dissemination due to disruption of NRP1/PlexA1 heterodimerisation and subsequent inhibition of the PlexA1 dependent Rho-GTPase. Employing MTP-PlexA1 in GBM cancer models revealed an anti-angiogenic activity largely accounting for its antitumor activity. Overall, this study identifies PlexA1 as a novel potential biomarker of GBM as well as a novel therapeutic target for which we have developed a specific potent inhibitor.

## RESULTS

### PlexA1 is a prognostic marker of GBM

**Figure 1:**
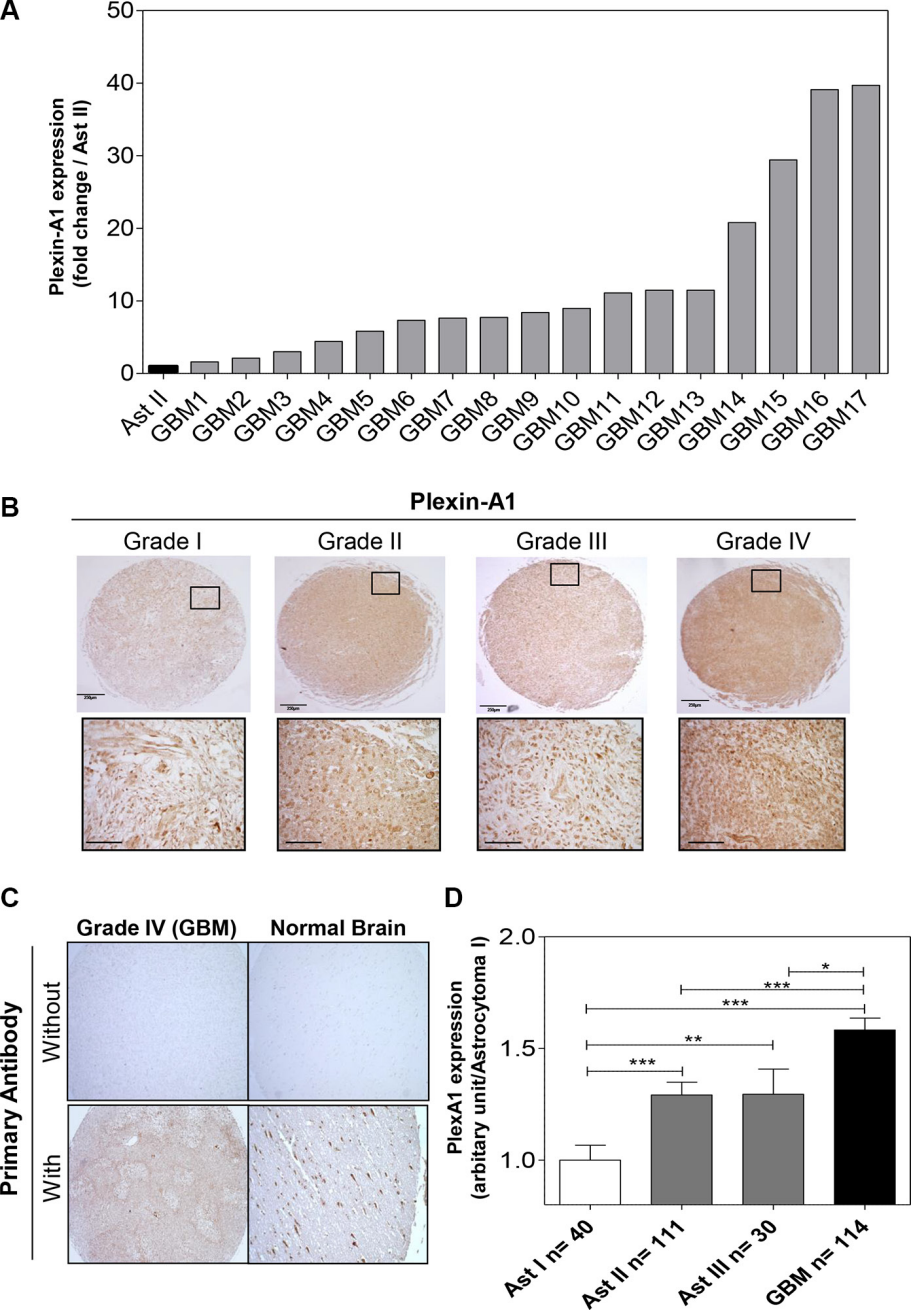
PlexA1 expression correlates with glioma severity (**A**) Q-RTPCR analysis of PlexA1 expression in 17 GBM biopsies compared to a low grade astrocytoma (grade II). (**B**) Tissue array analysis. Insets show higher magnification of each picture (scale bar: 40 μm). (**C**) Quality control experiments verifying the specificity of the signal in a GBM and a normal brain sample with (positive control) or without primary antibody (negative control). (**D**) Quantification of PlexA1 expression level according to the grade of the tumor, Ast I = Astrocytoma grade I, Ast II = Astrocytoma grade II, Astrocytoma grade III, GBM = Glioblastoma.

We first determined the expression of PlexA1 in our collection of 17 GBM RNA samples using Q-RTPCR. This revealed a systematic overexpression of PlexA1 ranging from 1.6- to 40-fold when compared to grade II astrocytoma (Figure 1A). To further explore the expression profile of PlexA1 we performed a tissue micro-array (US Biomax) on a total of 295 biopsies of patients with glioma (Figure 1B). Normal brain tissue served as positive control and negative control was performed by omitting primary antibody (Figure 1C). Quantitative analysis revealed a correlation between glioma grade and the level of PlexA1 expression. Grade II and grade III astrocytoma showed increased levels of PlexA1 being intermediate to grade I and IV (Figure 1D). To examine whether the high expression of PlexA1 in GBM may have a prognostic value we performed data mining of the Rembrandt repository collection [20] (Supplementary Figure S1). Our analysis of 385 annotated gliomas revealed that patients expressing the highest level of PlexA1 (above the median expression of PlexA1) had a reduced probability of survival (Median survival = 510 days) when compared to patients expressing lowest level of PlexA1 (below the median expression of PlexA1, median survival 689 days, *p* = 0.0018, log rank test). This large scale analysis confirmed the results obtained with the tissue array. Strikingly, when restricting the analysis to the group of GBM (grade IV) patients only (*n* = 181), the correlation between the high level of PlexA1 and a reduced survival was still significant. Median survival was 369 days for patients with expression above median while it reached 474.5 days for patients whose expression of PlexA1 was below the median (*p* = 0.0225, log rank test). Further analysis taking into account age or gender did not reveal additional information (data not shown). However, we were able to confirm this correlation of high expression of PlexA1 to poorest survival in an independent data set, the TCGA repository collection. In this collection of 499 GBM the median survival was 466 days for patients with the lowest PlexA1 (below the median expression) and 370 days for those with highest expression (above the median expression, *p* = 0.005, log-rank test, Supplementary Figure S1D).

### Molecular simulations analyzing PlexA1 and NRP1 TMD interactions

**Figure 2:**
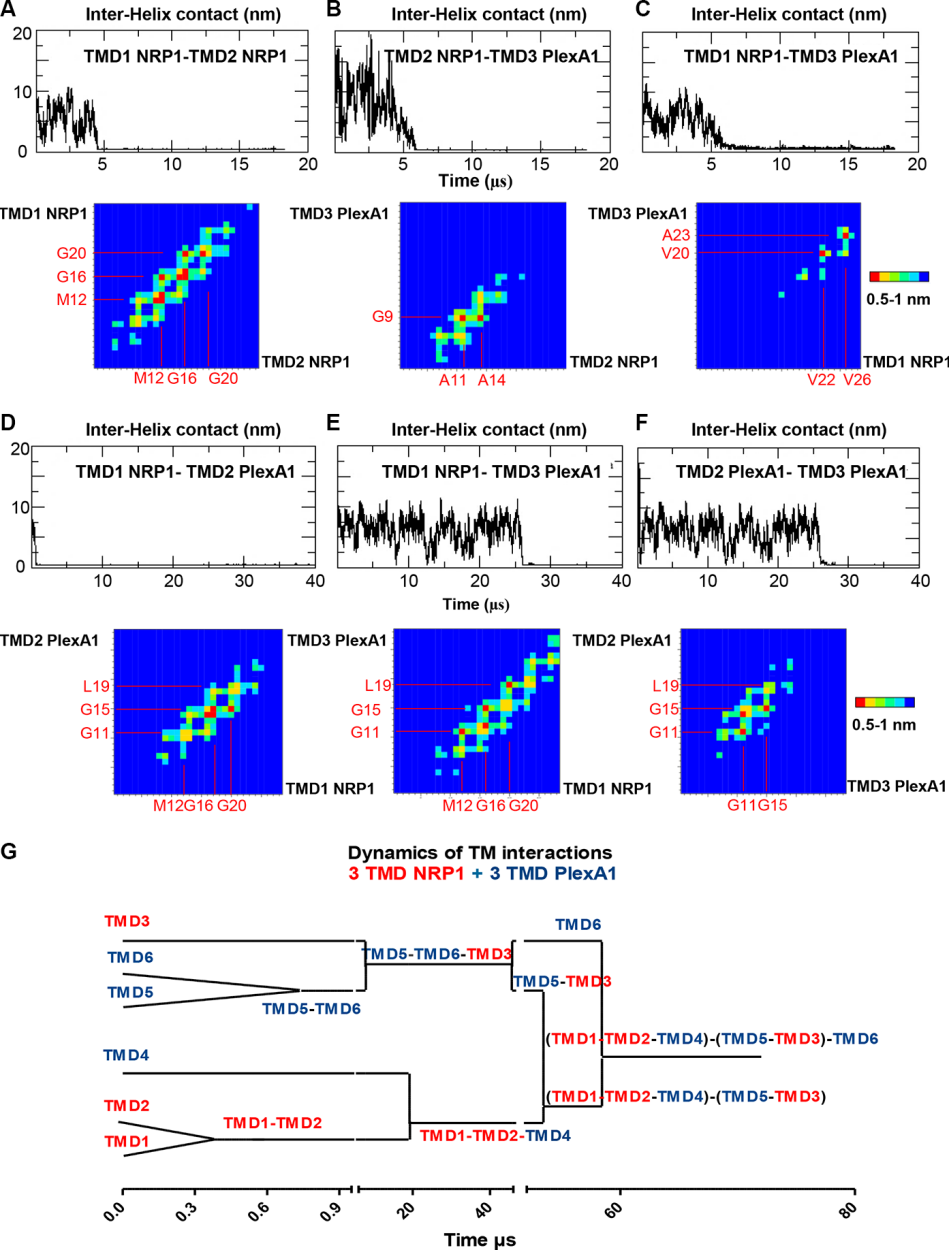
Dynamic of PlexA1 TMD interactions The graphs represent the inter helix distance between two monomers as a function of time. Contact maps exhibit the residue pairs (key interfacing residues) having the smallest distances between two helices backbones. The red squares correspond to the closest distances 0.5 nm < d < 0.6 nm and the blue squares correspond to the longest distances (d > 1 nm). The yellow, green and light blue squares correspond to intermediate distances 0.6 < d < 0.7, 0.8, 0.9 nm). (**A**–**F**) CG simulation including different compositions of NRP1 and PlexA1 TMDs. (**G**) Dynamics of the interactions between 3 NRP1 TMDs and 3 PlexA1 TMDs inserted in DOPC bilayer along a 72 μs simulation.

Previous results discovered using coarse grain simulation in a DOPC (Dioleoylphosphatidylcholine) membrane bilayer model that NRP1 and PlexA1 TM domains produce homo- and hetero-dimers [21] which had been confirmed in a biochemical assays [22]. Here, we extended this analysis by simulating multiple interactions between NRP1 and PlexA1 TM domains. The time required for the formation of the TMD1 NRP1-TMD2 NRP1 homodimer is not exceeding 5 μs (Figure 2A top, simulation 1). The two TM domains associated in right handed interactions with a crossing angle of −32° on average. The contact map (Figure 2A) showed symmetric and well-defined interacting motif M12xxxG16xxxG20 with closest distances around 0.5 nm. One μs later TMD3 PlexA1 approached TMD1 NRP1-TMD2 NRP1 homodimer to form a stable trimer until the end of the simulation (18 μs) (Figure 2B and 2C top). The crossing angles were +33° and +4° on average for the heterodimers TMD2 NRP1-TMD3 PlexA1 and TMD1 NRP1-TMD3 PlexA1 respectively. The TMD2 NRP1-TMD3 PlexA1 contact map exhibited the key interfacial residues A11 and A14 for TMD2 NRP1 and G9 for TMD3 PlexA1 (closest distances around 0.5 nm) (Figure 2B bottom). Milder contacts, with closest distances around 0.7 nm, were observed between TMD1 NRP1 (V22 and V26) and TMD3 PlexA1 (V20 and A23) (Figure 2C bottom). A representative conformation of this trimer is shown in Supplementary Figure S3A. When simulating the association of TMD1 NRP1-TMD2 PlexA1-TMD3 PlexA1 interaction (simulation 2) we very quickly (about 1 μs) observed the formation of a TMD1 NRP1-TMD2 PlexA1 heterodimer (Figure 2D top). The two TMD associated in right handed interactions with a crossing angle of −33° on average. Beyond 26 μs the TMD3 PlexA1 monomer diffused within the membrane and then joined the TMD1 NRP1-TMD2 PlexA1 heterodimer to form a stable trimer until the end of the simulation (40 μs) (Figure 2E and 2F top). The TMD1 NRP1-TMD3 PlexA1 heterodimer and TMD2 PlexA1-TMD3 PlexA1 homodimer associated in right handed interactions with crossing angles equal to −36° and −45° respectively. The contact maps exhibited the interacting motif M12xxxG16xxxG20 for TMD1 NRP1 and the key residues G11, G15 and L19 for TMD2 PlexA1 and TMD3 PlexA1. A representative conformation of this trimer is shown in Supplementary Figure S3B. Hence, a longer molecular dynamic simulation implicating 3 NRP1 TMD and 3 PlexA1 TMD (simulation 3) showed that interactions were highly dynamic and allowed transitions from hetero-dimers to trimeric complexes eventually interacting with each other (Figure 2G). Thus, the CG-MD simulations suggested that adding a synthetic peptide mimicking the TMD of PlexA1 could compete with naturally occurring TMD interactions thereby leading to signal transduction alteration as previously described [11].

### PlexA1 TMD exhibit specific interactions

**Figure 3:**
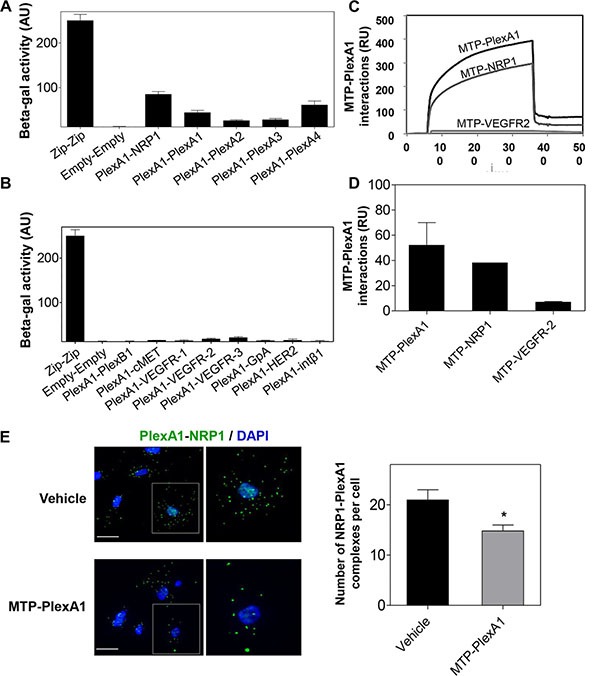
Specificity of the PlexA1 TMD interactions (**A**, **B**) BACTH assay demonstrating the positive or negative homo- or hetero dimerization of PlexA1 TMD with the TMD of putative co-receptors. “Zip sequence” is the positive control of dimerization and “Empty” is the negative control of this bacterial system. (**C**, **D**) SPR assay (Biacore) to quantify MTP-PlexA1 interactions with PLexA1, NRP1 and VEGFR2 TMDs. (**E**) Proximity ligation assay showing that MTP-PlexA1 (10^−7^ M) decreases the number of PLexA1-NRP1 interactions (green dots) in U373MG cells (scale bar = 20 μm).

To further explore the TMD interactions of PlexA1 we used a bacterial two-hybrid system for a more systematic analysis of TMD interactions [22]. TMD sequences of PlexA1 and various potential interacting partners including NRP1 and all other members of the Plexin-A family were co-expressed in the bacteria to define the hierarchy of the interactions. This interaction study confirmed computer simulations as demonstrated by a significant interaction between NRP1 and PlexA1 TMD (Figure 3A). The systematic analysis confirmed the homo-dimerization capacity of PlexA1 and also revealed positive interactions with the TMD of Plexin-A4 but weaker interactions with Plexin-A2 and Plexin-A3. The use of additional constructions encoding the TMD of Plexin-B1, cMET, VEGFR-1, VEGFR-2, VEGFR-3, GPA, HER2 or Integrin Beta-1 showed no significant interaction with PlexA1 TMD while all of these receptors contain GxxxG-like motifs thereby demonstrating the high specificity of the interacting profile of PlexA1 TMD (Figure 3B).

A surface plasmon resonance (SPR, Biacore) analysis was also conducted with a synthetic biotinylated PlexA1 peptide immobilized on the chip. This allowed us to confirm some of the results obtained by BACTH because the PlexA1 TMD mimetic peptide significantly interacted with NRP1 (chosen as a positive interaction according to the BACTH assay) but not with VEGFR-2 (chosen as a negative interaction from the BACTH results) (Figure 3C–3D). Due to the high hydrophobic nature and difficulty to produce TMD peptides we only performed this experiment with TMD peptides for which we had experience of successful synthesis.

### MTP-PlexA1 disrupts dimerization of PlexA1 with its co-receptors

**Figure 4:**
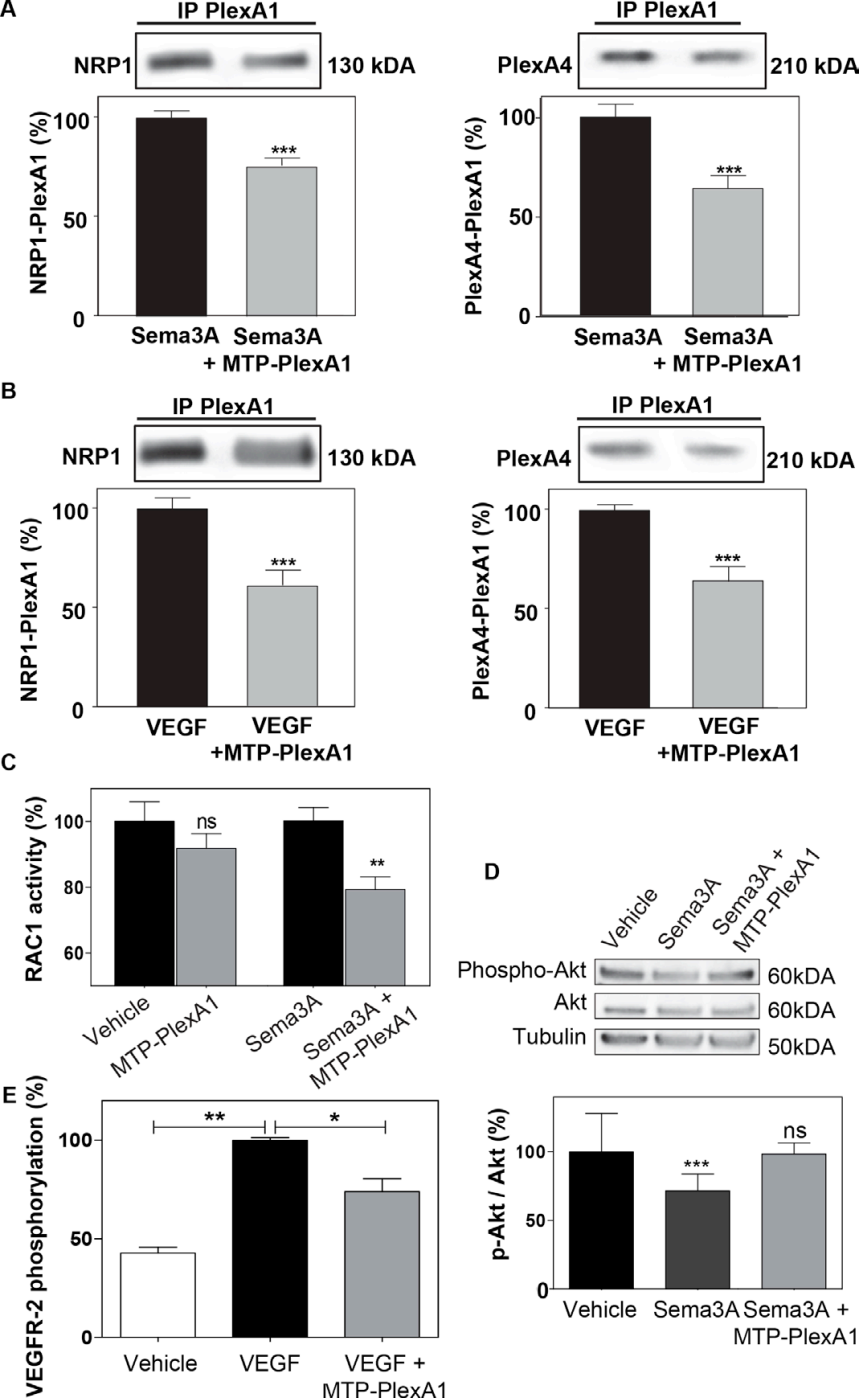
MTP-PlexA1 inhibits PlexA1 signaling (**A**) Representative immunoblotting images revealing the amount of NRP1 and PlexA4 co-immunoprecipitated with PlexA1 in U373MG cells stimulated with Sema3A (100 ng/ml) or Sema3A + MTP-PlexA1 (10^−7^ M) (A) or in cells stimulated with VEGF (100 ng/ml) or VEGF + MTP-PlexA1 (10^−7^M) (**B**). (**C**) Rac-1 activation assay in resting condition and after Sema3A-induced Rac-1 activation in U373 MG cells. (**D**) Western blot analysis of phosphorylated Akt, total Akt and tubulin in U373MG cells treated with the vehicle (LDS 72 μM), Sema3A (100 ng/ml) or Sema3A + MTP-PlexA1 (10^−7^ M). The lower panel is showing the quantification of the p-Akt/Akt ratio in the different conditions. (**E**) MTP-PlexA1 decreases VEGFR-2 phosphorylation induced by VEGF (100 ng/ml) in HUVEC cells.

To demonstrate that MTP-PlexA1 is able to alter receptor complex formation we performed an *in situ* proximity ligation assay (Duo-link system, Sigma-Aldrich) [23]. As seen in Figure 3E, we observed numerous NRP1/PlexA1 complexes at the cell surface (U373MG cells) in control conditions (vehicle treated cells). However, the addition of MTP-PlexA1 induced a 29.4% reduction of NRP1/PlexA1 complexes. Additional specificity controls were performed using a mutated version of MTP-PlexA1 (in which 3 glycines of the GxxxGxxxG motif were replaced by 3 valines or MTP-Neu, an anti-breast cancer peptide known to not interact with PlexA1) as described in Supplementary Figure S2. Moreover, we found that Sema3A-induced co-immunoprecipitation of NRP1 or Plexin-A4 with PlexA1 was significantly reduced (−24.6% for NRP1 and −35.5% for PlexA4) in the presence of MTP-PlexA1 (Figure 4A). Similar results were obtained when cells were exposed to VEGF (39.4% NRP1 and 35.4% PlexA4, Figure 4B). Altogether these results demonstrate that MTP-PlexA1 is able to reduce oligomerization of PlexA1 in resting conditions or in response to Sema3A or VEGF ligands.

### MTP-PlexA1 blocks the signaling capacity of PlexA1

PlexA1 is a Rho-GTPase activating protein. Consistently, it has been shown that in response to Sema3A, the Rho-GTPase Rac1 is activated and sequestered at the plasma membrane [24]. Here, using an ELISA-based assay we were able to show that MTP-PlexA1 significantly inhibited Sema3A-induced Rac1 activity while not affecting this Rho-GTPase activity in the absence of ligand (Figure 4C). Moreover, it has been shown that Sema3A inhibits Akt phosphorylation through binding of Rnd1 to PlexA1. Our data show that Sema3A-induced inhibition of Akt phosphorylation (−33%, Figure 4D) is blocked by the addition of MTP-PlexA1. Hence, a previous study suggested a pro-angiogenic effect of PlexA1 [25]. Indeed, when measuring the phosphorylation of VEGFR-2 using an ELISA assay we also found that MTP-PlexA1 significantly reduced VEGF-induced phosphorylation of VEGFR-2 (−26%, Figure 4E). From these experiments we concluded that MTP-PlexA1 is able to antagonize major signaling pathways of PlexA1 receptor.

### PlexA1 exhibits a pro-angiogenic activity that can be blocked by MTP-PlexA1

**Figure 5:**
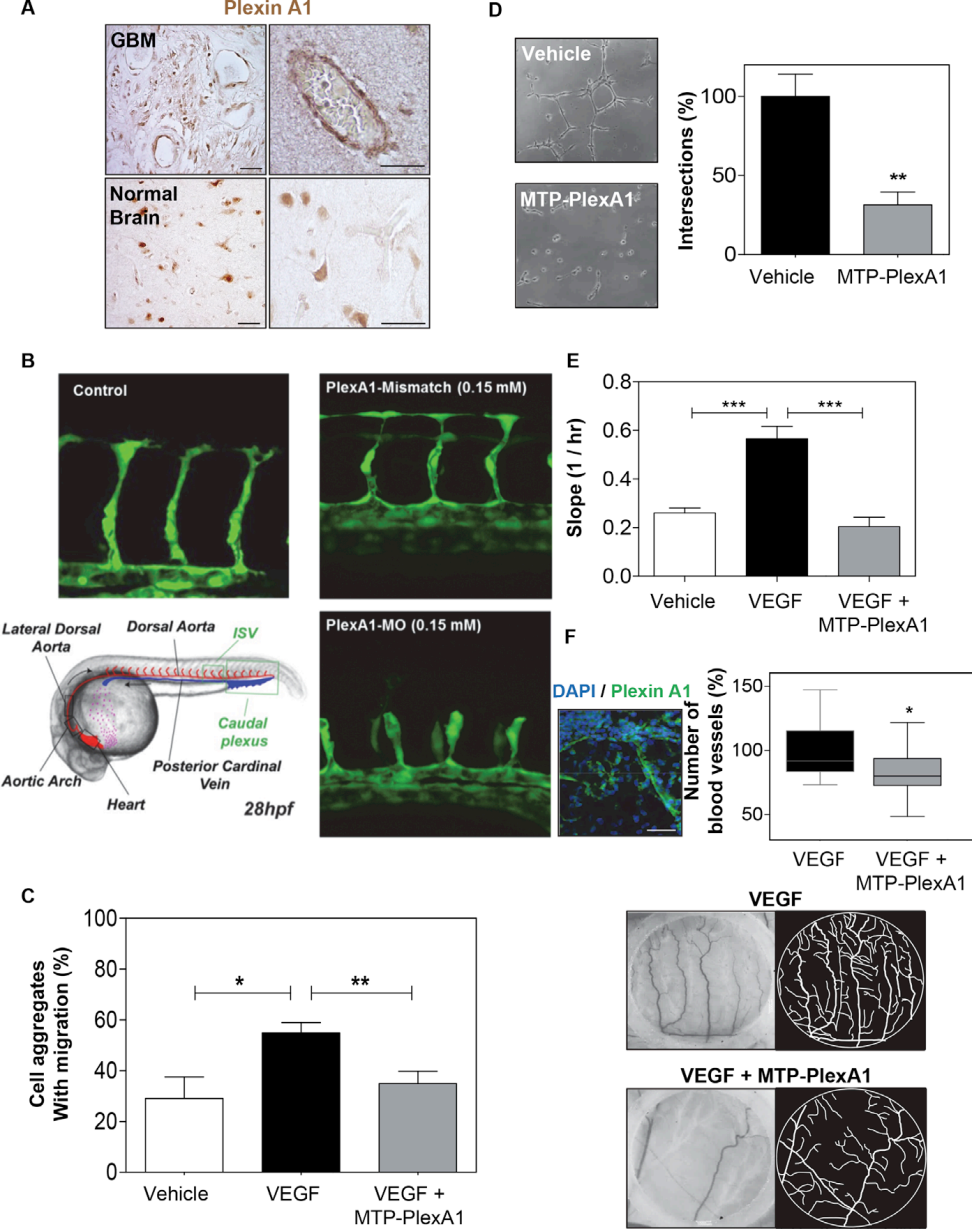
MTP-PlexA1 inhibits developmental and tumor-specific PlexA1 pro-angiogenic role (**A**) Microphotographs illustrating the expression of PlexA1 in vascular-like structures in human glioma tissues. (Scale bar: 40 μm). (**B**) Morpholino-based PlexA1 knockdown in Zebrafish *tg(kdrl:eGFP)* line. Representative images of control, Plexin-A1 mismatch and Pexin-A1 knockdown embryos (plxna1MO) are shown 28 hours post fertilization (28hpf). (**C**) Demonstration of the capacity of MTP-PlexA1 (10^−7^M) to block VEGF (100 ng/ml)-induced migration of HMEC cells from micro-aggregates growing in a 3D plasma clot. (**D**) Demonstration of MTP-PlexA1 anti angiogenic effect on HUVEC cells grown 5 h on a matrigel with 10^−7^M of the peptide or in control (vehicle treated) condition. (**E**) Live-monitoring of VEGF-induced HUVEC cell migration assay using the XCelligence system (transwell assay). (**F**) Microphotographs illustrating the expression of PlexA1 in blood vessels of the chick chorioallontoic membrane (upper left picture). Microphotographs (middle and lower left pictures) and camera lucida drawing (middle and lower right pictures) showing representative fields of observation after 24 h incubation with VEGF (100 ng/ml, +vehicle) or VEGF + MTP-PlexA1 (10^−7^ M). The number of VEGF-induced new blood vessels is shown in the graph.

We observed PlexA1 positive blood vessels in the tissue array of GBM samples (Figure 5A). Interestingly, blood vessels in control normal brain tissue were not expressing PlexA1. Additional experiments performed in the mouse brain showed that only developing embryonic but not mature adult blood vessels expressed PlexA1 (see Supplementary Figure S4). These data suggested a role of PlexA1 during developmental and pathological angiogenesis. To address this possibility we first used a transgenic zebra-fish model *tg(kdrl:eGFP)* highlighting the endothelial cells. This allowed us to assess the angiogenic activities *in vivo* by analyzing formation of intersegmental vessels (ISVs) after morpholino-based knockdown of the target. Indeed, using a morpholino sequence against PlexA1 previously characterized [19] we observed a significant number of abnormal angiogenic sprouts in ISVs when compared to controls (no injection of morpholino) or mismatch PlexA1 morpholino 28 hours post fertilization (Figure 5B). This result confirmed *in vivo* the importance of PlexA1 in blood vessel development. We next performed a 3D migration assay with HMEC spheroids grown in a plasma clot and showed that VEGFA-induced cell migration was abolished when adding the PlexA1 inhibitory peptide (Figure 5C). Moreover, using a pseudo-tube formation assay with human endothelial HUVEC cells grown on matrigel, we found that the addition of MTP-PlexA1 significantly blocked tube-like structure intersections thereby demonstrating a negative impact of the peptide on HUVEC migration (−68%, *p* = 0.0087) (Figure 5D). Interestingly, the use of the mutated version of MTP-PlexA1 confirmed the specificity of this anti-angiogenic effect because MTP-PlexA1mut was not able to reduce the number of tube-like structures (see Supplementary Figure S2B). This inhibition of VEGFA-induced HUVEC cell migration was confirmed in a live monitoring trans-well assay with MTP-PlexA1 (−63%, *p* = 0.0002; X-Celligence system, ACEA Biosciences, Figure 5E). Hence, to further validate the inhibition of the PlexA1 pro-angiogenic activity in the presence of MTP-PlexA1 we also analyzed the vascular development of the chorio-allantoic membrane of the chick embryo. In this assay we confirmed the expression of PlexA1 in developing blood vessels and we also showed that local deposition of MTP-PlexA1 significantly inhibited VEGFA-induced vascular growth and complexity (Figure 5F). Altogether, these results characterized the pro-angiogenic effect of PlexA1 and demonstrated that this effect can be fully antagonized with MTP-PlexA1.

### MTP-PlexA1 inhibits tumor growth

**Figure 6:**
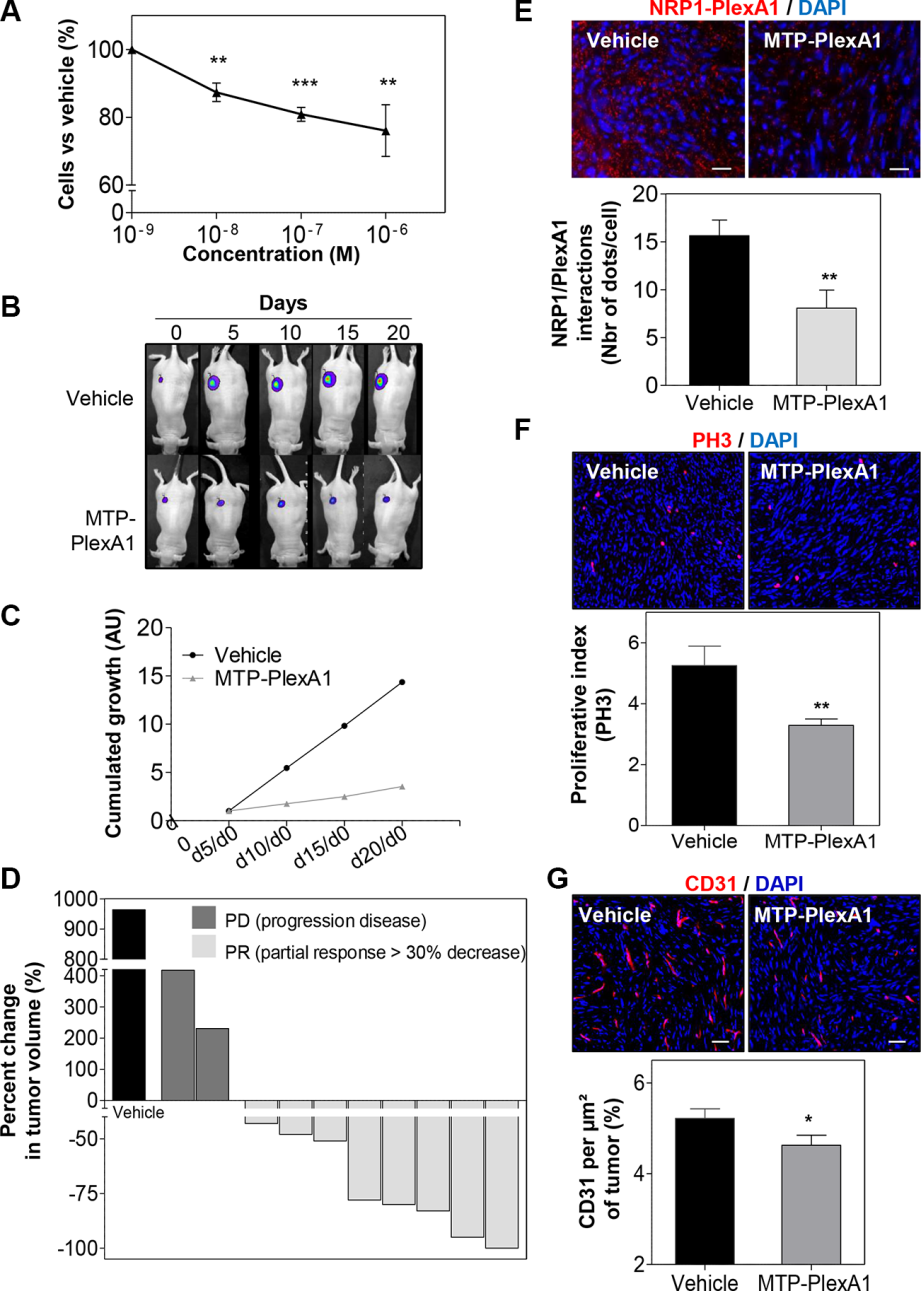
Demonstration of the anti-tumor effect of MTP-PlexA1 (**A**) MTT assay showing the dose-dependent anti-proliferative effect of MTP-PlexA1 after 24h culture of U118MG. (**B**) Representative photographs illustrating the kinetic of tumor growth over 20 days in the two experimental groups. (**C**) Cumulated growth curves of the tumors (UA: Arbitrary Unit). (**D**) Waterfall plot of best response representing the percent change in tumor volume of individual treated animals (grey bars, *n* = 10) compared to the average tumor volume increased determined in the control group (dark bars, *n* = 10). (**E**) Proximity ligation assay on tumor slices showing that MTP-PlexA1 (10^−7^ M) decreases the number of PlexA1-NRP1 interactions in U118MG cells. (**F**) Representative microphotographs and related quantification of PH3 positive cells in tumor slices (**G**) Representative microphotographs and related quantification of CD31 positive regions in tumor slices (% CD31 per μm² of tumors).

To address the biological consequences of MTP-PlexA1-mediated inhibition of PlexA1 we used the U118MG-Luc glioma cell line genetically engineered to express the luciferase reporter gene. The addition of MTP-PlexA1 to U118MG-Luc cells reduced proliferation in a dose dependent manner as measured in a MTT assay (Figure 6A). Cells were then grafted subcutaneously and tumor growth was monitored every 5 days for a total period of 20 days. As seen in Figure 6B, U118MG-Luc formed large tumors continuously growing in control conditions while tumor growth was dramatically slowed down in the animals receiving the therapeutic peptide every day (1 μg/kg). The quantitative analysis of the cumulated luminescent signal acquired all along the protocol confirmed the strong reduction of tumor development translating into a marked 75% reduction of the averaged luminescent signal at the end point (Figure 6C). The waterfall graph of best response [26] also showed that 2 out of 10 mice did not respond to the treatment. However, responses were strong for 8 out of 10 mice with some responses close to 100%, all of them being at least Partial Response (PR) (Figure 6D). In order to clarify the mechanism by which MTP-PlexA1 is exerting tumor growth inhibition we collected tumors and analyzed them histologically. When performing a proximity ligation assay on tumor tissue sections we could visualize a two-fold reduction of interactions of PlexA1 and NRP1 when mice had been treated with MTP-PlexA1 (−48.5%, *p* = 0.0051, Mann Whitney test, Figure 6E). We also found that the number of proliferative cells determined by counting PH3 positive cells on the whole surface of 5 sections per tumor was 37.3% (*p* = 0.0022, Mann Whitney test) decreased in treated animals compared to the control group (Figure 6F). Moreover, when determining the density of blood vessels using CD31 immunostaining we found a significant 11.4% reduction (*p* = 0.0436, Mann Whitney test) of tumor-associated blood vessels in the animal group receiving MTP-PlexA1 (Figure 6G). This part of the study demonstrated that inhibition of tumor growth *in vivo* can be explained by an anti-proliferative and an anti-angiogenic effect of MTP-PlexA1 mirroring the results of the *in vitro* assays. Hence, we performed an orthotropic grafting experiment to monitor the effect of MTP-PlexA1 on GBM tumors developing in their native microenvironment. While not strongly impacting on tumor volume (−14.5%, *p* = 0.54, Mann Whitney test) the intraperitoneal administration of MTP-PlexA1 (1 μg/kg) every 3 days for 3 weeks induced a significant inhibition of cell proliferation (−38.3% PH3 positive cells *p* = 0.0023, Mann Whitney test, Supplementary Figure S5A–S5B) and reduced angiogenesis (−14.3% *P* = 0.0378, Mann Whitney test, Supplementary Figure S5C–S5D) in tumors of MTP-PlexA1 treated mice in comparison to controls. A similar anti-tumor effect was also demonstrated in tumors upon grafting the other cell line U373MG (Supplementary Figure S6).

### MTP-PlexA1 inhibits cancer stem cells-dependent tumor growth

**Figure 7:**
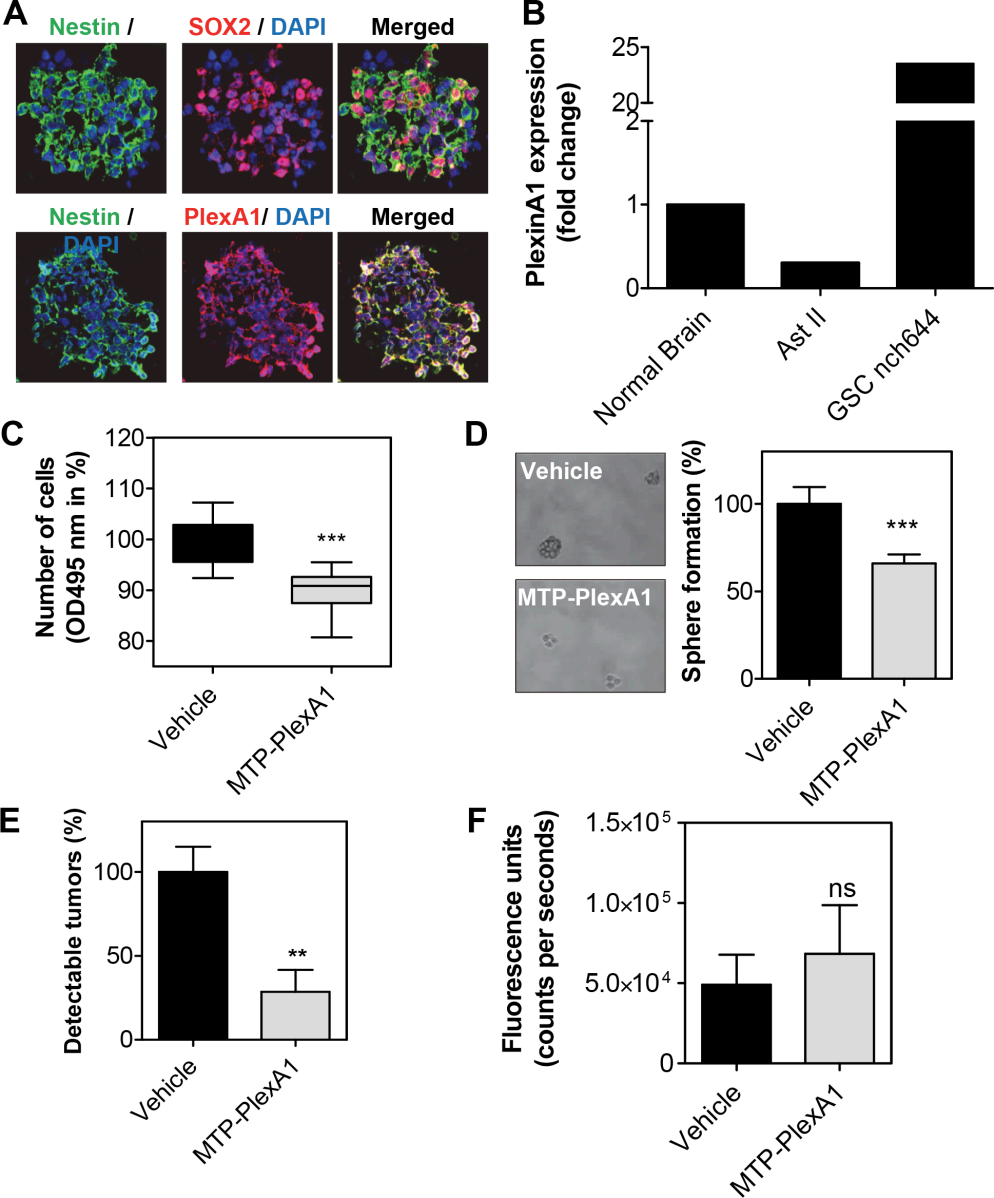
MTP-PlexinA1 inhibits cancer stem cells-dependent tumor growth (**A**) Expression of Nestin (green), Sox2 (red) in NCH644 glioma stem cells. PlexA1 receptor (red) is expressed in Nestin positive cells (green) (scale bar 100 μm). (**B**) Q-RTPCR analysis showing the expression of PlexA1 in normal brain, Astrocytoma II (Ast II) and NCH644. MTS proliferation assay (**C**) and sphere formation assay (**D**). (**E**) Detectable tumors (%) after *in vivo* grafting. (**F**) Fluorescence signal produced by tumors that were able to grow over the five days period in the vehicle treated cells (black bar) and MTP-PlexA1 treated cells (grey bar).

Mounting evidence supports a crucial role of cancer stem cells in the initiation or relapse of tumors [27]. This is particularly the case for gliomas [28]. Thus, we decided to evaluate to what extent MTP-PlexA1 would affect glioma stem cell (GSC) growth. To this end, we used the NCH644 human cell line derived from a patient biopsy [29]. As seen in Figure 7A, GSCs co-express PlexA1 and stem cell markers Nestin or Sox2. Moreover, Q-RTPCR analysis revealed that NCH644 cells exhibited strong overexpression of PlexA1 when compared to the level in normal brain (23 fold) or grade II astrocytoma (78 fold) (Figure 7B). Interestingly, MTP-PlexA1 reduced the proliferation capacity of the GSC grown in large populations as seen using a MTS assay (−10% *p* < 0.0001, Figure 7C). We next performed a sphere forming assay in which cells were plated into 96-well plates at a low density (30 cells per well) to monitor production of clones from individual cells growing as non-adherent gliomaspheres. A minimum of 30 cells per well was required to allow sufficient sphere production (composed of at least 8 cells) in a time frame compatible with drug testing. Visual control of sphere formation on a daily basis confirmed that cellular edifices were arising from single cells and not due to cell clumping. Strikingly, the addition of MTP-PlexA1 at a concentration of 10^−9^ M decreased the ability of GSC to form sphere by 33.3% (vehicle = 100% +/− 9.7%; MTP-PlexA1= 66.7% +/− 5.2, *p* = 0.0015, Student *T* test) thereby demonstrating the capacity of the peptide to inhibit GSC clonal expansion (Figure 7D). To address whether this inhibitory effect is sufficient to impede tumor development *in vivo* we performed a xenograft experiment. NCH644 GCS were grafted in the flank of nude mice in a way to obtain two bilateral tumors derived from either control cells without treatment or from cells that had been pre-incubated with MTP-PlexA1. This was done using a fluorescent version of the NCH644 cell line (mCherry-NCH644) allowing live monitoring of tumor appearance (Figure 7E). We choose to monitor tumor development over a 5 days period of time as a compromise to reach the size of detectable tumors while conserving significant inhibitory activity of MTP-PlexA1 (at least for the first three days as previously shown for equivalent membrane targeting peptides [12]). Because we failed to detect tumors when grafting the cells in the brain for sensitivity reasons, this experiment was conducted in a subcutaneous localization. Strikingly, with control cells we observed the development of 14 tumors in the 20 grafted mice (Figure 7E). However, GCS pre-incubated with the peptides only rarely gave rise to detectable tumors (4 out of 20 mice, −71.4%, *p* = 0.0032, Mann Whitney test). However, the pre-incubation of the cells with the peptide was not sufficient to block the proliferation of the cells over the 5 days period because the size of the few tumors that were able to grow was comparable to the one of the control group (*p* = 0.4258, Mann Withney test, Figure 7F).

## DISCUSSION

While confirming that targeting the transmembrane domain of membrane receptors offers a credible alternative to other existing drugs targeting intra or extra cellular domains of membrane receptors, our study identifies novel functions for PlexA1 with fundamental novel and clinical relevance.

The identification of suitable biomarkers is complicated in GBM by the short range of patient survival, the great heterogeneity as well as an incomplete understanding of the physiopathology of these tumors. For example, the IDH1 mutation appears to be a prognostic marker (with better outcome) for GBM [30] and EGFR amplification is frequent in GBM (25% to 33%, [31]). The MGMT methylation is also considered as a prognostic tool and a predictive marker of drug efficacy since it is correlated with a better response to alkylating agents such as temozolomide [32]. Here, we found that the expression of PlexA1 correlated with the grade of glioma defining three groups with low (grade I), medium (Grade II/III) and high PlexA1 expression in high grade glioma (Grade IV, glioblastoma). The prognostic value of PlexA1 has already been described for patients with pancreatic tumors [33]. Our results now suggest that PlexA1 expression level may also be considered as a potential diagnostic and prognostic marker in glioblastoma. In the present study, a striking feature of PlexA1 expression was the staining of tumor associated blood vessels in patient biopsies similarly to gastric cancer where PlexA1 is associated with microvessel density [34]. Intriguingly, we failed to detect PlexA1 in blood vessels in human normal brain or in adult mouse brain. The expression of PlexA1 in blood vessels was restricted to developmental and tumor angiogenesis. Thus, because angiogenesis is a marker of cancer severity [35] and more precisely of glioma malignancy [36], the prognostic value of PlexA1 may be the consequence of this expression in tumor-associated blood vessels. In the present study both *in vitro* and *in vivo* assays showed a clear pro-angiogenic role of PlexA1. This is consistent with previous work that showed inhibition of tube formation by HUVEC cells upon knocked-down of PlexA1 [25]. Because no study reports the direct binding of VEGF to PlexA1, PlexA1-mediated endothelial cell migration and blood vessel formation requires the involvement of VEGF binding partners and/or VEGFR modulators. The results of our co-immunoprecipitation experiment showed in the presence of MTP-PlexinA1 a reduction of PlexinA1-NRP1 and PlexinA1-PlexinA4 interaction, two receptors involved in glioma-associated tumor angiogenesis [25]. We however cannot exclude that additional pro-angiogenic ligands such as PDGF, FGF, TGF-b1, that have been shown to be linked to NRP1 [37–39], may also recruit PlexA1 directly or indirectly through the formation of specific receptor complexes. Coarse grain simulations revealed the dynamic association of NRP1 and PlexA1 TMDs being capable of homodimerization, heterodimerization, oligomerization and/or transitions from one status to the other. This highly dynamic behavior is probably the source of a wide range of interaction capabilities of PlexA1. The role of TMD in the association and stabilization of membrane anchored receptor is becoming well-described. This is demonstrated for several families of receptors including TLR [10], integrins [8] or tyrosine kinase [40]. Very recently, it has been shown that the activation of EGFR requires an N-terminal interaction between the transmembrane helices [41, 42]. These studies clearly showed that both TMD and juxtamembrane domain influence extracellular domain of EGFR receptor. Thus, besides the importance of extracellular domain interactions in regulating ligand binding and receptor association, TMD should now be also considered as crucial regulators of receptor activity. The contact maps we created from simulations revealed the existence of multiple potential interfaces with unexpected amino acids (such as the G11xxxG15xxxL19) favoring the different types of interactions between NRP1 and PlexA1 with adapted crossing angles. PlexA1 TMD contains long series of 6 glycines (G9, G11–G15) generating two GxxxG like motifs being on different faces of the helix therefore creating at least two opposite surfaces for interactions. Related TMD interactions are very specific because PlexB1, c-MET, VEGFR-1, −2, −3, HER-2, Integrin-β1 TMD exhibited no significant interaction with PlexA1 TMD. Thus, the existence of a GxxxG motif is not sufficient to trigger an interaction. Rather, the whole sequence is defining the specificity of interactions. Hence, it remains to be determined the exact fraction of peptide reaching the tumors (heterotopic or brain orthotopic tumors) to define the optimal dosage of the peptide. Moreover, combination therapies with standard of care strategies open novel opportunities. The most striking result was obtained when using the patient derived cancer stem cell line NCH644 [29]. Besides the high level of PlexA1 expression in glioma stem cells, we also found that MTP-PlexA1 inhibited sphere formation and proliferation. This is indicating that blocking PlexA1 directly inhibited the stem cell renewal capability in a range translating into a marked inhibition of tumor production *in vivo*.

In conclusion, we had identified PlexA1 as a diagnostic and prognostic marker of GBM. We also described the developmental or tumor associated pro-angiogenic activity of this receptor that also promotes tumor cell proliferation and migration. Furthermore we demonstrated that the transmembrane domain of this receptor regulates PlexA1 oligomerization and can be antagonized by a peptide mimicking the native sequence. Hence, this peptide appears as a good drug candidate to interfere with cancer stem cells and angiogenesis thus slowing down GBM growth.

## MATERIALS AND METHODS

### Cell culture

U373MG (08061901) and U118MG are human GBM cell lines obtained from the ECACC and the ATCC respectively. HUVEC (Human Umbilical Vein Endothelial Cell) provided by PromoCell (C-12200) and HMEC-1 (Human Microvascular Endothelial Cell-1) provided by Dr Ellen van Obberghen-Schilling (Institute of Biology Valrose, Nice). Details of culture are presented in Supplementary Method section.

### Tissue array and glioblastoma biopsies

Slides of human brain gliomas tissue arrays were obtained from US Biomax (BS17016a/GL2083a/GL806b/GL803a/CNS801). The detailed immunocytochemical procedure is provided in Supplementary Method section. Samples from patients diagnosed with GBM were provided by the Neurosurgery department of Hautepierre hospital and collected by the Centre de Ressources Biologiques (CRB). This collection was approved by the French Ministry of Health and received authorization number DC-2009-1016. All samples were anonymized for research. Total RNA was extracted after cell dissociation of human GBM surgical specimens immediately after resection. RNAs of grade II Astrocytoma were purchased from Clinisciences (CR562205). Details of the Q-RTPCR analysis are provided in Supplementary Method section.

### Modeling of TM interaction and MTP-PlexA1

Coarse-grained molecular dynamics (CG-MD) simulations were used to investigate the dynamics of the association of several NRP1 and PLXA1 TM domains when embedded in a DOPC (1,2-di-oleoyl-sn-glycero-3-phosphocholine) lipid bilayer. The GROMACS software package (version 4.5.4) [14, 15] was used. The TM peptides, the lipids and the water CG particles were described with the MARTINI force-field version 2.1 [16–18]. A detailed procedure of the system setup and analysis is provided in Supplementary Method section.

### Angiogenesis in the zebra fish

For knockdown of PlexA1 in the zebra fish we used morpholino oligonucleotides described by [19]. MO (5′-GCCACATATCTGCACTGGTCCTTGA-3′) was injected at the one-cell stage. Animals (Tg(flk1:egfp)) were incubated at 28.5°C for 5 hours before treatment with 1-phenyl-2-thiourea (PTU) to prevent pigment formation. Zebrafish embryos were staged, anesthetized with 0.02% tricaine solution, and mounted in drops of 0.8% low-melting point agarose (Sigma Aldrich). They were imaged at 28 hpf using a confocal microscope: both intersegmental vessels and the caudal plexus regions were imaged.

### Heterotopic xenografts

Experiments were performed according to the Guide for Care and Use of Laboratory Animals (E67-6-482-21) and the European Directive with approval of the regional ethical committee (Reference AL/55/62/02/13). Heterotopic grafts were produced by injecting 106 cells in the flank of pathogen-free NMRI nude mice (Janvier, Le Genest Saint Isle, France). Intraperitoneal administration of vehicle (LDS 72 μM) or MTP-PlexA1 treatment (10–7 M) were done every days during 20 days when the tumors reached a minimal volume of 100 mm3. For the experiment conducted with cancer stem cell line NCH644mCherry, we performed bilateral grafting of 10^6^ cells on each flank of 20 nude mice without treatment or pre-incubated for 1 hour with MTP-PlexA1 at 10–7 M. After 5 days, the fluorescence emission of developing tumors was recorded using the NightOwl system (Berthold) using appropriate excitation and emission filters (580 nm/620 nm respectively).

### Chick embryonic chorio-allantoic membrane assay

The CAM assay was performed using Leghorn eggs. After 4 days incubation at 37°C, the shells were opened and the embryos were transferred to a Petri dish. The CAMs were grown for additional 4 days before deposition of silicone reservoirs in a region containing one large vessel. Reservoirs were filled with 20 μl VEGF165 solution (20 μg/ml) or a combination of VEGF165 (20 μg/ml) + MTP-PlexA1 (10–7 M). After 24 h incubation, micro- photographs were taken for quantification of vessel growth. Camera lucida drawing was systematically performed to allow better counting of blood vessels and sprouting; the entire analysis being performed in blind conditions.

### Sphere formation assay

Cells were stained with an orange fluorescent dye (CMRA orange, Molecular Probe) to facilitate monitoring of individual cell and sphere counting. Cells were grown into a 96-well plate at a density of 30 cells per well containing 50 μl of culture medium (containing MTP-PlexA1 at a concentration of 10–9 M or 0.072 μM vehicle) during 4 days. The sphere formation rate was established by counting the number of spheres per well. Cellular edifices were considered as spheres only when composed of at least 8 cells. The sphere forming rate was calculated by dividing the number of sphere at day 4 per the real number of cells at day one in a given well.

### Statistics

Statistical analyses were performed using Mann Whitney test (for sample *n* < 30), Chi square analysis (for qualitative data including proportion of phosphorylated/unphosphorylated receptors and co-immunoprecipitation experiments), Extra sum of square test (for curve trend analysis) or Log-rank test (for survival analysis) using GraphPad software (USA). *P*-values are given in the figure legends, and values of *p* < 0.05 were considered to be statistically significant. Normal distribution of the values was checked using GraphPad software (USA). A minimum of three independent experiments was performed for *in vitro* assays.

## SUPPLEMENTARY MATERIALS FIGURES AND TABLE




