# Supplementary material for: Inhibition of PlexA1-mediated brain tumor growth and tumor-associated angiogenesis using a transmembrane domain targeting peptide
Source: Oncotarget. 2016 Aug 5;7(36):57851–65. doi: 10.18632/oncotarget.11072 (PMC5295395; doi:10.18632/oncotarget.11072)
Supplement: Supplementary file 1 [file oncotarget-07-57851-s001.pdf]

Inhibition of PlexA1-mediated brain tumor growth and tumor-associated angiogenesis using a transmembrane domain targeting peptide

Supplementary Materials

Molecular simulation: system setup

NRP1 and PlexA1 TMD sequences were taken from the native potential sequences given in the Uniprot database (http://www.uniprot.org/). The length of the TM domains was adjusted in order to design a 28 amino acid sequence by adding a few residues on each side. For the sake of clarity, the TMD sequences have been re-numbered 1 to 28. The CG helical structures of the TM domains were modeled as ideal helices and were considered electrically neutral.

Three bilayer systems (2 trimers and one hexamer) were prepared. One is composed of 3 TMDs including 2 NRP1 TMDs and 1 PlexA1 TMD, a second one is composed of 3 TMDs including 1 NRP1 TMD and 2 PlexA1 TMDs. The third one includes 3 NRP1 TMDs and 3 PlexA1 TMDs.

The TMD peptides were inserted in a large equilibrated bilayer including DOPC (1, 2-di-oleoyl-sn-glycero-3-phosphocholine) initially built from the initial pre-equilibrated DOPC bilayer taken from the MARTINI web site (http://md.chem.rug.nl/cgmartini/). The X, Y, Z dimensions of the bilayer were 18.7, 19.2, 9.8 nm respectively and contained 1068 lipids and 15460 water molecules.

For these three systems, the peptides were inserted in a parallel manner, the helical axis being parallel to the bilayer normal (Z axis). Each peptide was randomly rotated about the helix axis thus leading to a random orientation of GxxxG motifs of one monomer relative to the other ones. During lipid insertion a small number of lipids and water molecules (a few tens depending on the systems) were removed from the sites of peptide insertion. Within the bilayer, the initial distance between the TMD peptides is about 6 nm from each other.

Each of these initial systems was energy minimized and equilibrated before performing MD simulations at 300 K. The temperature was coupled to a thermostat using a time constant of 1 ps applied separately to the peptides, the lipids and the water groups using a Berendsen algorithm [49]. The pressure was coupled using a semi-isotropic scheme (lateral and perpendicular pressures being coupled independently at 1 bar with a coupling time of 0.5 ps and a compressibility of 4.5 × 10–5 bar-1). A time step of 0.040 ps was used for the production step. Lennard Jones and Coulombic interactions were shifted to zero between 0.9 and 1.2 nm and 0.0 and 1.2 nm respectively. These same conditions were applied for the 1 µs equilibration of the pure DOPC bilayer.

The duration of the simulations differ depending of the system studied and the simulation time was sufficiently long to observe a stable assembly of the peptides. Simulations were stopped when stable aggregates were observed. Then, simulation 1 (trimer 2 NRP1 TMDs + 1 PlexA1 TMD), simulation 2 (trimer 1 NRP1 TMD + 2 PlexA1 TMDs) and simulation 3 (hexamer 3 NRP1 TMDs + 3 PlexA1 TMDs) were performed over 18 µs, 40 µs and 72 µs respectively.

Molecular simulation: system analyses

Analyses were performed using tools provided in the Gromacs package [41], Inter-helix distances (distance between the center of mass of the helix backbones and inter-helix contact distances (minimal distance between the helix backbones) were calculated. An associated state of TM helices corresponds to a contact distance less than 0.5 nm. The longest duration of an associate state was chosen to calculate the contact matrices representing, on average, the smallest distances between the backbone beads of the residue pairs. Helix-helix interfaces were then extracted from the contact matrices.

Retrospective analysis of *plexA1* expression in human gliomas

For the “Rembrandt dataset” [17], data (raw CEL files, Affymetrix HG-U133_Plus_2 arrays) were downloaded from caArray (https://caintegrator.nci.nih.gov/caintegrator/workspace.action; NCI, USA) 226 GBM and 28 controls. Raw data were RMA normalized using the Expression Console software (Affymetrix). In each cohort, GBM patients were stratified using the median value of *Plxna1* expression as a cutoff, using the *Plxna1* 221538_s_at probe set. GBM with a *Plxna1* expression above the median of expression in the cohort were

assigned to the "highest *Plxna1*" group and conversely those with a *Plxna1* expression below the median to the "lowest *Plxna1*" group. A Kaplan-Meier survival analysis was then performed using the log-rank test to assess the significance of survival differences between the two groups (GraphPad Prism).

For the "TCGA dataset" [50, 51], data were downloaded from the TCGA data portal (https://tcga-data.nci.nih.gov/tcga/tcgaHome2.jsp; NCI, USA). RMA normalized gene expression profiles measured with Affymetrix U133A arrays were used. Clinical annotations were available for 499 patients to perform KaplanMeier survival analyses.

Cell culture

U373MG and U118MG are human GBM cell lines obtained from the ECACC and the ATCC respectively. Cells are grown at 37°C, 5% CO2 in Dulbecco's modified Eagle medium (DMEM, GIBCO) supplemented with 10% fetal calf serum (FCS) (GIBCO), 1% penicillin, 100 μg/ml streptomycin (Sigma). HUVEC (Human Umbilical Vein Endothelial Cell) provided from PromoCell (C-12200) and HMEC-1 (Human Microvascular Endothelial Cell-1) provided from Dr Ellen van Obberghen-Schilling (Institute of Biology Valrose, Nice) are grown in the Endothelial Cell Growth Medium Kit (C-22110) supplemented with Fetal Calf Serum at 0.02 ml/ml, Endothelial Cell Growth Supplement 0.004 ml/ml, Epidermal Growth Factor (recombinant human) 0.1 ng/ml, Basic Fibroblast Growth Factor (recombinant human) 1 ng/ml, Heparin 90 μg/ml, Hydrocortisone 1 μg / ml. The NCH644 GSC is from a collection of GSC lines established from patient glioblastome multiforme biopsies and was kindly provided by Christel Herold-Mende (University of Heidelberg, Heidelberg, Germany). These cells form spheres and are cultured in neurobasal-A medium (Gibco) with 2% of B27 neural supplement (Gibco), 2 mM of L-glutamine (Gibco), 1% of Penicillin-Streptomycin (Sigma) and 20 ng/ml of hEGF (Strahtmann AG) and bFGF (Millipore).

Tissue array analysis

Slides of human brain gliomas tissue arrays were obtained from US Biomax (BS17016a/GL2083a/GL806b /GL803a/CNS801). For immunocytochemical detection of PlexA1, the paraffin sections (5 μm thickness) were de-waxed and rehydrated through 100% toluene (2 washes of 15 minutes) then 100% alcohol (2 washes of 15 minutes), 95% alcohol, 90% alcohol, 70% alcohol and water (1 wash 10 minutes respectively) then an antigen retrieval step was done in sodium citrate buffer (10 mM, pH = 6, Sigma #CO759) 2 × 10 min in the microwave. After incubation with blocking solution (PBS + 5% FCS) for 20 minutes, the primary rabbit anti-PlexA1 antibody (Abcam, ab32960-100) was added to the sections in blocking solution at 4°C overnight at a dilution of 1/100. An extensive washing was then completed (3 × 5 min in PBS) before incubation with the secondary antibody (Goat anti-rabbit, Vector Laboratories, #PI-1000) at a dilution of 1/200 in PBS for 1 hour at room temperature. The tissue was rinsed with PBS (3 × 5 minutes) and then the endogenous peroxidases were blocked with 1% H2O2 solution (Sigma, #H1009) in methanol solution during 20 minutes. This later solution was washed out and amplification of the signal was achieved using the ABC peroxidase solution (Elit Vectastain kit, PK-6100). DAB was finally added (kit from Vector laboratories). Lastly sections were dehydrated in 70% alcohol, 90% alcohol, 95% alcohol, 100% alcohol with rapid dips in each bath before final wash in toluene for 5 minutes and mounting in Eukitt (Sigma, #03989).

Q-RTPCR on glioblastoma biopsies

Samples from patients diagnosed with GBM were provided by the Neurosurgery service of Hautepierre hospital at Strasbourg. Total RNA was extracted after cell dissociation of human GBM surgical specimens immediately after resection. RNA of grade II Astrocytoma were purchased from Clinisciences (CR562205). To analyze the RNA level of PlexA1, RNA was extracted with TriReagent solution according to manufacturer's instruction (Molecular Research Center Inc., Euromedex). RNA was treated with DNase I (Invitrogen) and reverse transcribed using the High Capacity cDNA RT Kit (Life Technologies). Quantitative reverse transcriptase polymerase chain reaction (RTQ-PCR) was performed using the TaqMan Gene Expression Master Mix (Life Technologies) using the 7500 Real time PCR System (Life Technologies) following the manufacturer's protocol. TaqMan PlexA1 probe (Hs.432329) was used to quantify human PlexA1 mRNA. Samples were analyzed using 2 μl cDNA. All data were normalized to the GAPDH human reference gene using TaqMan GAPDH probe (Hs99999905). The calculation of PlexA1 RNA quantity in the GBM biopsies was effectuated as the following: Δct(GBMx) = ct(PlexA1) – ct(GAPDH), mRNA quantity = 2^(-Δct(PlexA1)). Each value was normalized by the 2^(-Δct(Astrocytoma II PlexA1).

Biacore analysis procedure

Binding experiments were performed by surface plasmon resonance measurements on a Biacore 2000 instrument (Biacore Inc.) at 20°C. Streptavidin was immobilized at high surface densities (~9,000 response units) on an activated CM5 chip using standard amine-coupling procedures as described by the manufacturer. Streptavidin was injected at a concentration of 250 μg/ml

in 20 mM sodium acetate (pH 4.5) at a flow rate of 5 µl/ min until desired surface densities, measured in response units, were reached. Unreacted groups were blocked by injecting 1 M ethanolamine. To perform peptide-peptide interaction assays, biotinylated PlexA1 peptide and other peptides were diluted in a buffer containing 10 mm HEPES, pH 7.4, 150 mM NaCl, 0.005% (v/v) surfactant P20 and 3 mM LDAO to maintain peptides in solution. The biotinylated-MTP-PlexA1 peptide was immobilized onto the sensor surface of a Biacore streptavidin sensor chip prior addition of the different interacting peptides injected at a flow rate of 5 µl/min. The coating of biotinylated-MTP-PlexA1 was accomplished by using a mixture of biotinylated-MTP-PlexA1 and an excess of non-biotinylated peptide to favor the dimeric biot-MTP-PlexA1/MTP-PlexA1 status prior fixation. After fixation, the elution buffer containing detergent removed non-biotinylated peptides rendering the chip ready for analysis with monomeric biot-MTP-PlexA1. Blank surfaces were used for background corrections. Injections of 30 µl 1 M LDS were used to regenerate surfaces between two binding experiments. We used steady state analysis to estimate kinetic parameters of peptide-peptide interaction.

Western blot and immunoprecipitation

U373MG cells were seeded at a density of 600 000 cells per well in a 6-well plate and serum starved overnight. Before protein extraction, the cells were treated either with 10^{-7} M of MTP-PlexA1 or Vehicle (LDS, 72 µM) during 1 h. Stimulation was done with 100ng/ml Semaphorin 3A for 30 min or with 100 ng/ml VEGF for 10 minutes. After a rapid PBS wash, the protein sample preparation was completed with PBS + Triton 0.1% supplemented with protease inhibitor (Roche, #11836145001) and 5 mM of Na ortho-vanadate. Immunoprecipitations were done with anti-human PlexA1 antibody (R&D, MAB6536), µMACS Protein G MicroBeads (Miltenyi biotec, 130-071-101) and matching micro-columns (Miltenyi biotec, 130-042-701). Following steps were done according to the manufacturer's instructions. Proteins were resolved in a 4–5% SDS/PAGE gel and transferred onto a nitrocellulose membrane (Whatman). The blots were soaked in blocking solution (TBS/0.1% tween/5% milk) for 1 h at RT. First antibodies (Rabbit anti-phospho-akt, Rabbit anti-akt, Cell Signaling #4060 and #9272 respectively) and mouse anti-tubulin α (Cell Signaling #2144) were incubated overnight at 4°C (+ anti-NRP1, anti-PlexA4 Abcam). After several washes (3 times 5 minutes, TBS/tween 0.1%), secondary antibodies (anti-rabbit-HRP, GE Healthcare, #NA934V and anti-mouse-HRP, GE Healthcare, #NXA931) were incubated 1h a RT in TBS/1% tween /5% BSA. The revelation step was performed using streptavidin-biotinylated Horseradish Peroxidase complex (Amersham #RPN1051) according to the manufacturer instructions. Images of the immuno-blots were acquired and analyzed thanks to the GENE GNOME apparatus (Syngene Bio Imaging, UK).

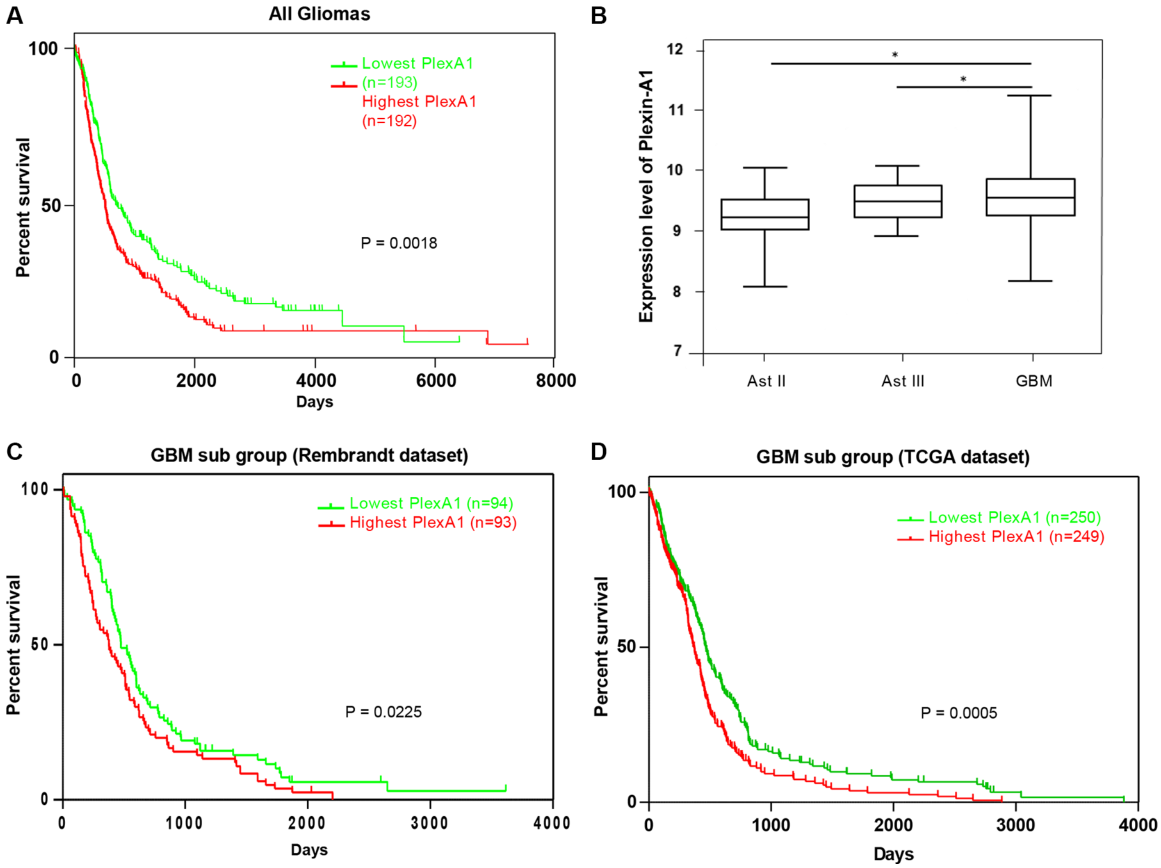

Supplementary Figure S1: *(***A)** PlexA1 gene expression correlates with poor survival of glioma patients (Rembrandt collection, *n* = 285). (**B**) PlexA1 gene expression correlates with the grade of glioma being the most expressed in high grade GBM (Rembrandt collection). (**C**) PlexA1 gene expression correlates with patient survival when restricting the analysis to the GBM subgroup (Rembrandt collection, *n* = 191). (**D**) PlexA1 gene expression correlates with patient survival when restricting the analysis to the GBM subgroup (TCGA collection, *n* = 499). The log-rank test was used to assess the significance of survival differences.

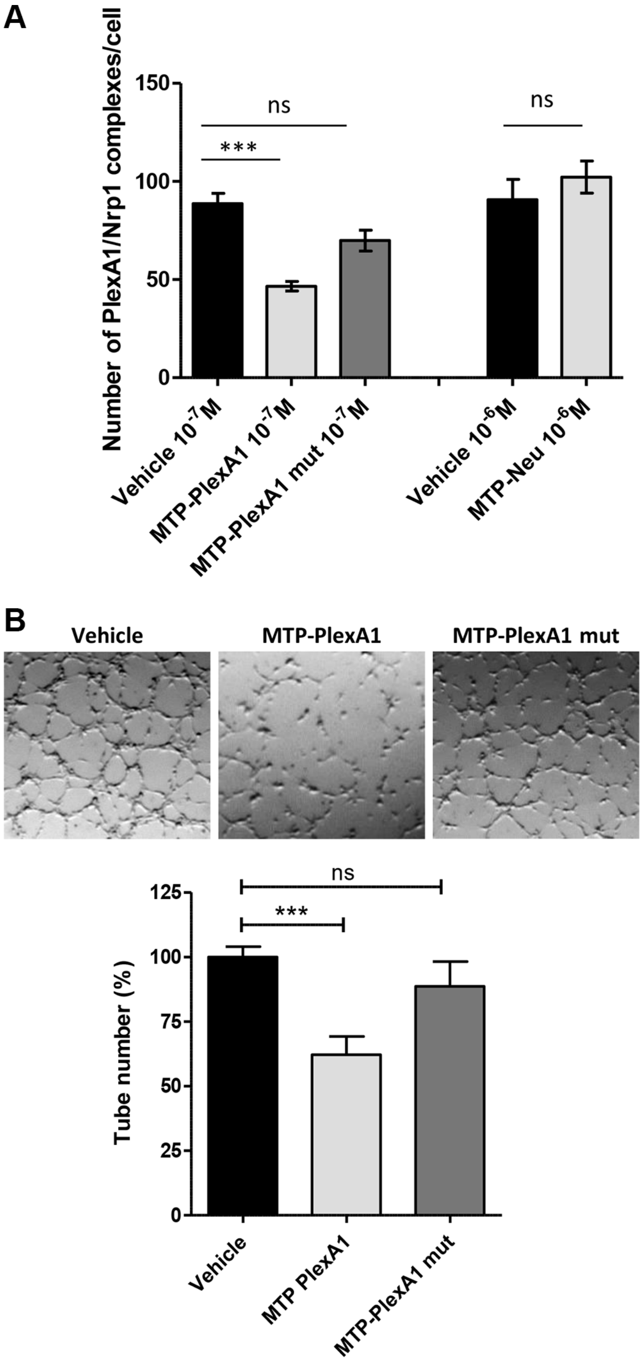

Supplementary Figure S2: Specificity controls were performed using a mutated version of MTP-PlexA1 called MTP-PlexA1mut exhibiting a triple mutation of glycines replaced by valines (Sequence: TLPAIVVIGGVGVLLL LVIVAVLIAYKRK). Such mutations have been previously shown to be sufficient to lose activity of NRP1 antagonist membrane domain targeting peptide (Roth et al., 2008 Mol Biol Cell). We also used MTP-Neu, a transmembrane domain targeting peptide known to not interact with NRP1 or PlexA1 (Sequence: TFIIATVEGVLLFLILVVVVGILIKRR as described previously in Arpel et al., 2014, Cell Reports). Indeed, when performing proximity ligation assay, NRP1/PlexA1 dimers were only significantly disrupted by wild type peptide but not by mutated MTP-PlexA1mut peptide or unrelated MTP-Neu peptide (A) Notably, the MTP-Neu peptide was used here at 10–6 M, a concentration previously shown to produce maximal antagonist activity of the peptide. Additionally, we also repeated the in vitro angiogenic assay on matrigel using HUVEC cells. Here again, MTP-PlexA1mut was not able to decrease tube like structure as compared to MTP-PlexA1. These experiments confirmed the specificity of MTP-PlexA1.

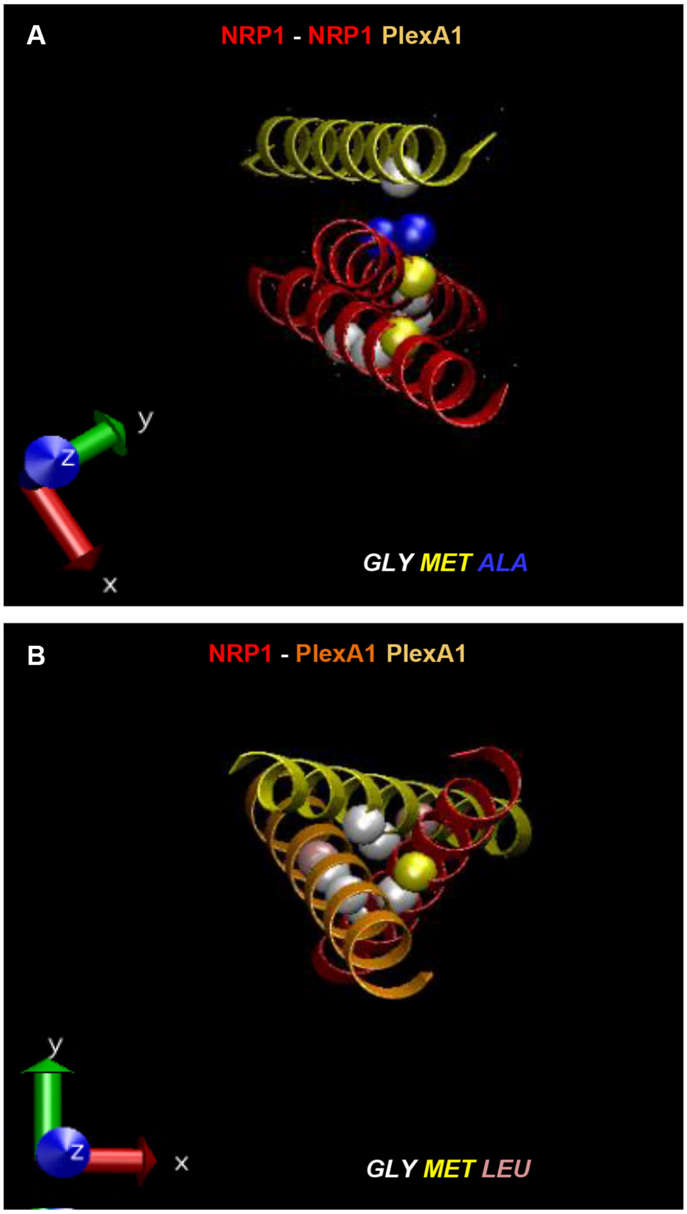

Supplementary Figure S3: 3D molecular model illustrating a PlexA1/NRP1 trimer. The key interfacing residues depicted on the contact maps (Figure 2) are highlighted in bead representations with a color code (GLY in white, MET in Yellow, ALA in blue and LEU in pink). The NRP1 TM monomers are figured in red. The PlexA1 TM monomers are figured in orange or in yellow. (**A**) Trimer of 2 NRP1 TM domains and 1 PlexA1 TM domain. (**B**) Trimer of 1 NRP1 TM domain, 2 PlexA1 TM domains.

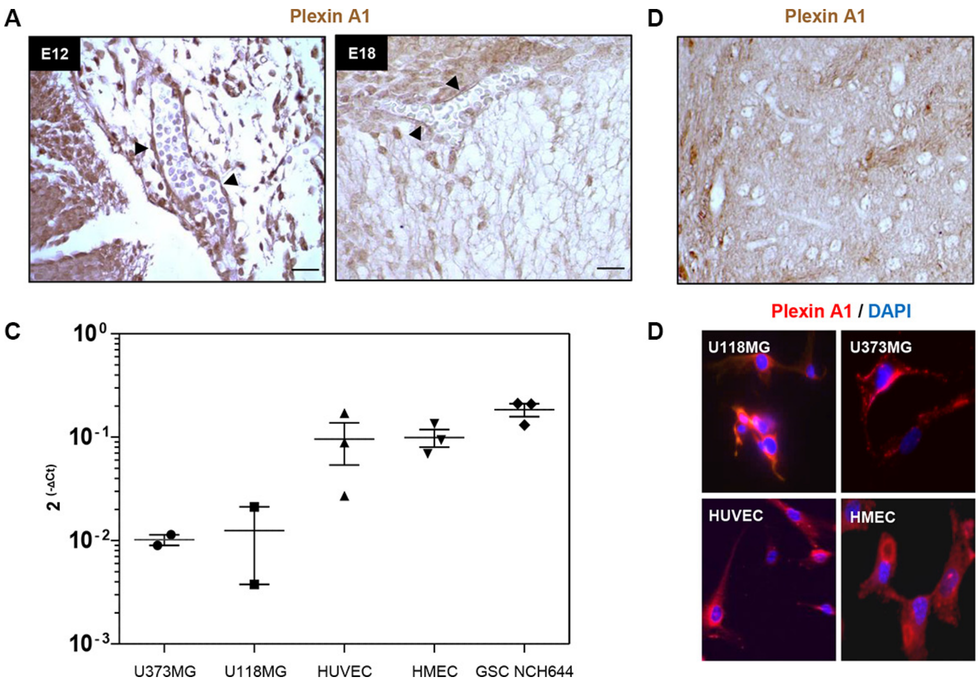

Supplementary Figure S4: (A) Microphotographs illustrating the expression of PlexA1 in vascular-like structures in the mouse brain during development (E12 and E18). Black arrow heads highlight blood vessels. Scale bar: 40 µm (**B**) Microphotograph illustrating the lack of PlexA1 expression in vascular-like structures in adult mouse brain. Black arrow heads highlight blood vessels. Scale bar: 40 µm. (**C**) RT-QPCR analysis demonstrating the expression level of PlexA1 expression in all cell lines used in this paper (U373MG, U118MG, HUVEC, HMEC and NCH644 cell lines). (**D**) Confirmation of the expression of PlexA1 at the protein level in U373MG, U118MG, HUVEC, HMEC cell line. The expression in NCH644 is shown in figure 7.

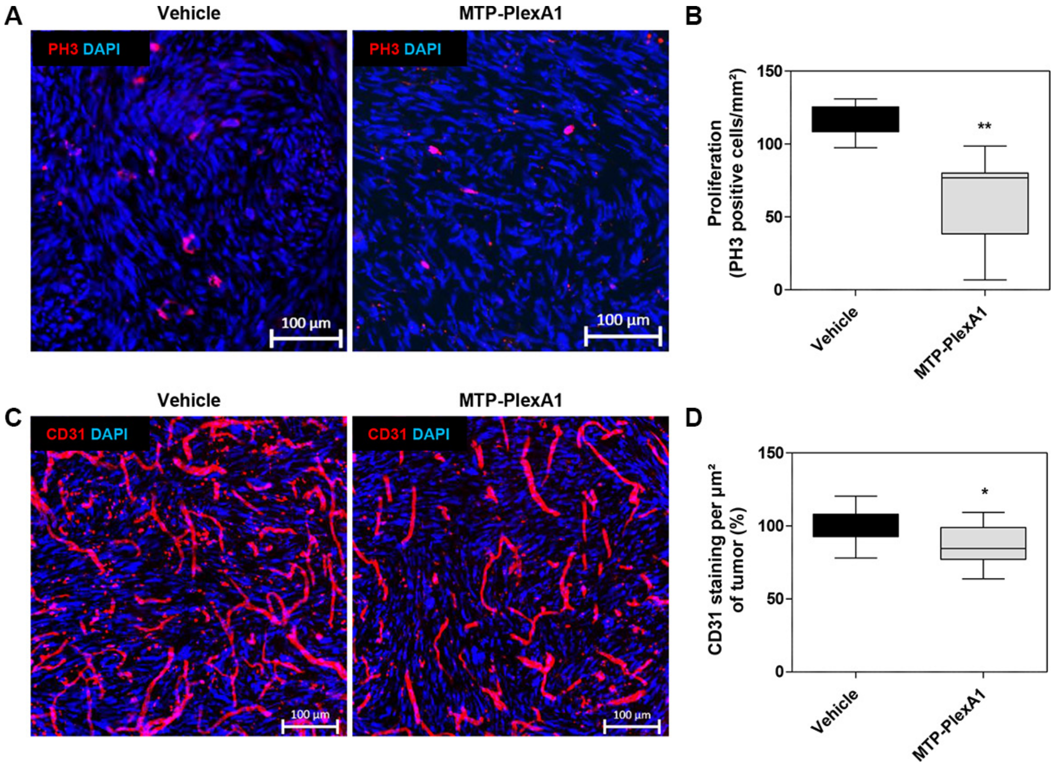

Supplementary Figure S5: We performed an orthotropic grafting experiment to monitor the effect of MTP-PlexA1 on GBM tumors developing in their native microenvironment. All procedures using animals are submitted to the Animal Care and Use Committee of AL/54/61/02/13. Immunocompromised mice are anesthetized with isoflurane 3% (BAXTER), under 0.5 L O_2, and received a subcutaneous injection of ketofen 1% 1.5 mL/Kg (MERIAL). The scalp is incised and 100 000 cells of the dissociated GBM biopsy were engrafted with a 2 µL Hamilton syringe (HARVARD APPARATUS; HAM-88400) mounted on a stereotaxic set up (HARVARD APPARATUS) to inject cells at the following coordinates from the bregma x: −2 mm; y: +1 mm; z: −3.5 mm at a speed of 0.66 µl/min, 5 minutes after the syringe introduction. After injection, the syringe is kept in place for 5 minutes, and then raised for 1 mm per minute. The scalp is sutured with 7.5 mm Michel stainless steel wound clips (A75, PERFECT). Animals are allowed to recover under a heat lamp several minutes before they were returned to their cage. After 10 days of tumor development, mice are randomized in 2 groups of treatment. The first group is treated with LDS (n = 12, IP, 72 µM) and the second group with MTP-PlexA1 (n = 12, IP, 1.5 µg/kg) 3 times per week. The administration volume is 100 µL. On the day of termination (21 days after the first treatment), all mice are sacrificed by cervical dislocation, brains are removed (24 total samples), and included in cryo-embedding media OCT® and stored at −80°C. Brains are cut in 40 µm sections with a cryostat (LEICA CM 3050S), mounted on blade (Superfrost Super Plus), and stored at −20°C. In order to visualize the tumor, brain sections are thawed in distilled water and Stained with 2% diluted Giemsa's staining (SUBRA; RAL diagnostic 320310–1000) in distilled water at 37°C for 2 hours. Sections are then rinsed in distilled water, incubated in 0.5% aqueous acetic acid (SIGMA; 33209–12) for 15 seconds, rapidly dehydrated in 70, 95 and 100% ETOH baths, in toluene (VWR Chemicals; 28676.297) and mounted under a cover slips (KNITTEL GLASS). Tumor volume is calculated by the addition of every partial volume: [$V_{total} = \Sigma$ partial volumes $= \Sigma\, d_1 \times (S1 + S2)/2 + d_2$ x $(S2 + S3)/2 + \ldots + d_n$ x $(S_n + S_{n+1})$]. All the surfaces are obtained by surrounding the tumor limits on every brain slices with ZEN software. The immunostaining procedure used to quantify proliferation and vascularization of the tumor is described in the method section.While not strongly impacting tumor volume (−14.5%, p = 0.54, Mann Whitney test) the intraperitoneal administration of MTP-PlexA1 induced a significant inhibition of cell proliferation (−38.3% PH3 positive cells p = 0.0023, Mann Whitney test, Supplementary Figure S5A–S5B) and angiogenesis (−14.3% p = 0.0378, Mann Whitney test, Supplementary Figure S5C–S5D) in treated tumors.

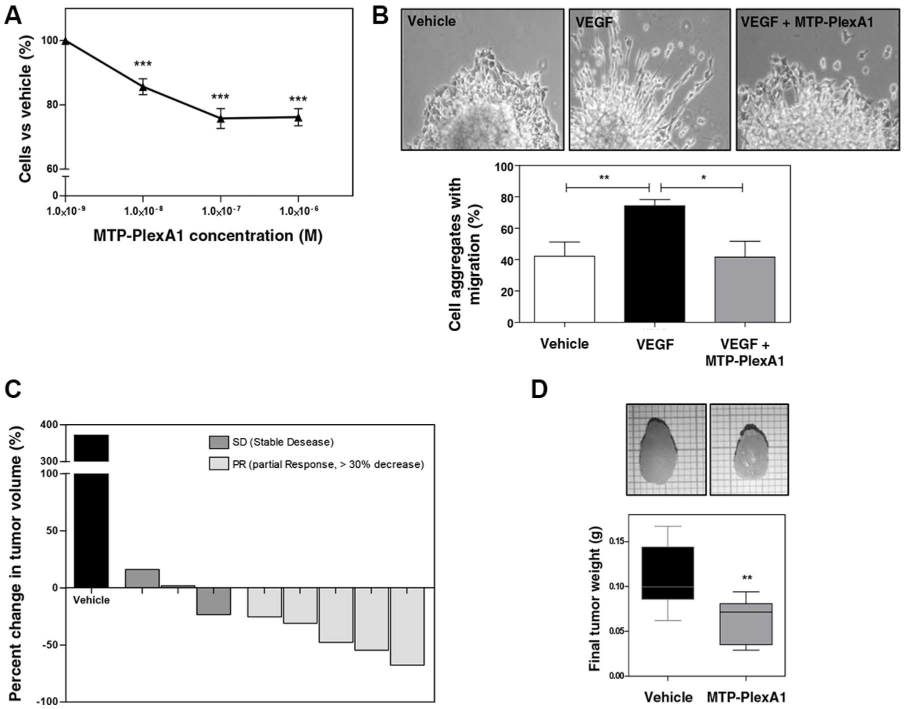

Supplementary Figure S6: The anti-tumor effect of MTP-PlxA1 was verified in U373MG human GBM line. We found a dose-dependent reduction of U373MG cell proliferation reaching a maximal effect at 10^{-7} M (–25%, $p < 0.0001$, Mann Whitney test). Because PlexA1 is involved in cell migration we also performed a 3D migration assay. U373MG cells were grown as cell aggregates (micro tumor-like spheroids) for 24 h in plasma clot with or without VEGF. Under these conditions, VEGF (100 ng/ml) induced tumor cell migration that formed cell chains invading the 3D substrate as previously described (12). Strikingly, VEGF-induced cell migration was abolished when MTP-PlexA1 at 10^{-7} M was added to the cultures. Hence, while confirming the proliferative and pro-migratory functions of PlexA1, this part of the study exemplified the therapeutic potential of MTP-PlexA1 because this peptide was able to block two major mechanisms of tumor progression. Indeed, when grafting U373MG cells in the flank of nude mice to produce tumors we found that the daily intraperitoneal administration of MTP-PlexA1 (10^{-7} M) significantly reduced endpoint tumor weight (−44%, $p = 0.0044$). A waterfall plot of best response using RECIST criteria as described by (24) showed that 100% of the mice responded to the treatment with 37.5% SD (Stable Disease) and 62.5% PR (Partial Response).

Supplementary Table S1: Sequences of synthesized peptides (MTP-x) and TMD sequences evaluated in the BACTH assay

Peptides	Sequences
TMD Plexin A1 / MTP-PlexA1	TLPAIVGIGGGGGLLLLVIVAVLIAYKRK
TMD Neuropilin-1 / MTP-NRP1	ILITIIAMSALGVLLGAVCGVVLYRKR
TMD Plexin A2	TLPAIVSIAAGGSLLLIIVIIVLIAYKRK
TMD Plexin A3	TLPAMMGLAAGGGLLLLAITAVLVAYKRK
TMD Plexin A4	SLPAIVSIAVAGGLLIIFIVAVLIAYKRK
TMD Plexin B1	AAQVGLGVGTSLLALGVIIIVLMYRRK
TMD c-Met	NFTGLIAGVVSISTALLLLLGFFLWLKKR
TMD VEGFR-1	NLELITLCTCVAATLFWLLLTLFIRKK
TMD VEGFR-2	IIILVGTAVIAMFFWLLLVIILRTVKRK
TMD VEGFR-3	SMEIVILVGTGVIAVFFWVLLLLIFCNRRK
TMD GpA	IEITLIIFGVMAGVIGTILLISYGIRRK
TMD HER2	ISIISAVVGILLVVVLGVVFGILIKRR
TMD Integrin β-1	IIPIVAGVVAGIVLIGLALLLIWKLLKR

Supplementary Video1: Molecular dynamics analysis simulating the interaction between 2 Plexin-A1 (yellow) and 1 Neuropilin-1 (red) transmembrane domains. **This movie correspond to 40 µs simulation in DOPC (1,2-di-oleoyl-sn-glycero-3-phosphocholine) lipid bilayer. The left part is showing a lateral view and the right part a top view. (See Supplementary_Video1)**
